# Seasonal dynamics of the macrophyte test species *Myriophyllum spicatum* over two years in experimental ditches for population modeling application in risk assessment

**DOI:** 10.1002/ieam.4553

**Published:** 2021-12-03

**Authors:** Gertie H. P. Arts, Jasper van Smeden, Marieke F. Wolters, J. Dick M. Belgers, Arrienne M. Matser, Udo Hommen, Eric Bruns, Simon Heine, Andreas Solga, Seamus Taylor

**Affiliations:** ^1^ Environmental Risk Assessment Wageningen University and Research Wageningen The Netherlands; ^2^ Fraunhofer Institute for Molecular Biology and Applied Ecology IME Schmallenberg Germany; ^3^ Bayer AG Monheim Germany; ^4^ Adama Agricultural Solutions UK Ltd. Reading UK

**Keywords:** Field experiment, Growth rate, Model parameters, *Myriophyllum spicatum*, Risk assessment

## Abstract

*Myriophyllum spicatum* is a sediment‐rooted, aquatic macrophyte growing submerged, with a wide geographical distribution and high ecological relevance in freshwater ecosystems. It is used in testing and risk assessment for pesticides in water and sediment. Population models enable effects measured under laboratory conditions to be extrapolated to effects expected in the field with time‐variable environmental factors including exposure. These models are a promising tool in higher‐tier risk assessments. However, there is a lack of data on the seasonal dynamics of *M. spicatum*, which is needed to test model predictions of typical population dynamics in the field. To generate such data, a two‐year study was set up in outdoor experimental systems from May 2017 to May 2019. The growth of *M. spicatum* was monitored in 0.2025 m^2^ plant baskets installed in an experimental ditch. Parameters monitored included biomass (fresh weight [FW] and dry weight [DW]), shoot length, seasonal short‐term growth rates of shoots, relevant environmental parameters, and weather data. The results showed a clear seasonal pattern of biomass and shoot length and their variability. *M. spicatum* reached a maximum total shoot length (TSL) of 279 m m^−2^ and a maximum standing crop above‐ground DW of 262 g m^−2^. Periodical growth rates reached up to 0.072, 0.095, and 0.085 day^−1^ for total length, FW, and DW, respectively. Multivariate regression revealed that pH (as a surrogate for the availability of carbon species) and water temperature could explain a significant proportion of the variability in *M. spicatum* growth rates (*p* < 0.05). This study has provided an ecologically relevant data set on seasonal population dynamics representative of shallow freshwater ecosystems, which can be used to test and refine population models for use in chemical risk assessment and ecosystem management. *Integr Environ Assess Manag* 2022;18:1375–1386. © 2021 The Authors. *Integrated Environmental Assessment and Management* published by Wiley Periodicals LLC on behalf of Society of Environmental Toxicology & Chemistry (SETAC).

## Key Points

- This study has generated a time‐series of seasonal dynamics for the growth of *M. spicatum* over two years under environmental conditions found in temperate regions to be used to develop and test population models for *Myriophyllum spicatum*.
- *Myriophyllum spicatum* showed a clear seasonal pattern of biomass and shoot length and of their variability (increasing in summer and decreasing in winter).
- Multiple regression modeling revealed that water temperature and pH (the latter as surrogate for the available carbon species) explained a significant part of the variability in *Myriophyllum* growth rates (*p*‐values < 0.05).
- Over the first four experimental months in summer, exponential functions yielded a better fit for the growth of *Myriophyllum spicatum* than linear growth functions.

## INTRODUCTION

Aquatic macrophytes form the basis of many food webs in aquatic ecosystems (Wetzel, 2001). Because of their important role in sustaining the functioning and diversity of the aquatic community, macrophytes are considered a key group. Hence, they are among the organisms used in the risk assessment of plant protection products, particularly of herbicides (EC, 2013), and are considered key drivers for setting specific protection goals (EFSA PPR, 2013). As aquatic macrophytes cover a wide range of different growth forms, the European Food Safety Authority (EFSA) (2013) and European Commission (EC) (2013) adopted the recommendation by Maltby et al. (2010) that not only the free‐floating standard laboratory test species *Lemna* sp. but also other macrophytes need to be considered in the risk assessment. If *Lemna* is found not to be sensitive to compounds with a herbicidal mode of action (e.g., in the case of auxins) or if exposure via sediment and/or pore water is of concern, the sediment‐rooted, submerged macrophyte *Myriophyllum spicatum* L. has been suggested as a suitable test species (Maltby et al., 2010).

*M. spicatum* belongs to the family of Haloragaceae and is a dicotyledonous, sediment‐rooted, vascular, submersed aquatic plant. It is native to Europe, Asia, and North Africa, and was introduced in the United States and Canada around 1940, where it is now considered an invasive species. This species offers several advantages as an additional test species in the aquatic risk assessment: it has an Eurasian distribution pattern (Cook, 1985) and is widespread in the temperate zone. It is among the most sensitive macrophyte species for several herbicides (Giddings et al., 2013), and has been proven to be an appropriate test species (Knauer et al., 2008) for assessing the risks of both water and sediment exposure (Burešová et al., 2013; Diepens et al., 2014). For all these good reasons, two Organisation for Economic Co‐operation and Development (OECD) test guidelines were developed for this species (OECD, 2014a, 2014b).

*M. spicatum* is a perennial plant that forms a dense fibrous root crown, from which numerous shoots grow towards the water surface (Smith & Barko, 1990). The species overwinters as unexpanded shoots attached to rootstocks and does not form specialized overwintering structures such as turions (Grace & Wetzel, 1978). Shoots growing from sediment form a canopy of leaves and branches close to the water surface (Aiken et al., 1979; Smith & Barko, 1990). The leaves are finely dissected and grow in whorls of four around the stem at each node. The plant forms a short, emergent inflorescence, composed of pollen‐forming flowers on top and seed‐producing flowers below, which are wind‐pollinated. Stems are long, slender, branching, and hairless, and become leafless toward the base (Adams et al., 1974). *M. spicatum* does not only show seasonal dynamics in biomass development and stoloniferous expansion (Madsen, 1997; Madsen & Smith, 1997; Riis et al., 2009), but also displays a seasonal cycle in storage of carbohydrates (Madsen, 1991). This cycle reaches a peak in late summer or fall and stored carbohydrates are depleted in spring. This seasonal cycle might influence macrophyte sensitivity, but this has never been investigated.

To analyze and predict the effects of pesticides under more realistic conditions, for example, under time‐variable exposure, TK–TD (toxicokinetic–toxicodynamic) and population models are considered by the regulatory community to be a promising tool in the higher‐tier assessment box (EFSA PPR Panel, 2013, 2018). The TK–TD models can be used as virtual laboratory tests for refined exposure analysis, in line with the tier 2C approach according to EFSA (2013, 2018). They can be combined with more complex population models to assess the potential effects under field conditions and over longer time scales. The potential use of such models to address the variety of exposure profiles predicted, for example, by the FOCUS step 3 and four models was shown during the MODELINK workshop (Hommen et al., 2016).

Until now, *M. spicatum* population models (Best & Boyd, 1999; Best et al., 2001; Heine et al., 2014) could only be tested with limited data sets derived from relatively short‐term laboratory, microcosm, and mesocosm studies or short‐term monitoring studies. However, evaluation and validation of models are critical for the acceptance of model predictions in a regulatory context (Augusiak et al., 2014). It is necessary to demonstrate that the models are able to adequately simulate population dynamics observed in the field, and EFSA (2018) reported that growth data for *M. spicatum* under natural conditions is currently lacking.

Given these gaps in our knowledge and the absence of multiseasonal time series, the aim of the present project was to provide a data set of seasonal dynamics of *M. spicatum*, including periods of growth during spring, abundance at the potential carrying capacity of aquatic ecosystems due to density dependence when *Myriophyllum* shoots reach the water surface and expected declines of abundance and biomass in autumn and winter. This data set is intended to allow testing and refinement of *M. spicatum* population models for application in the risk assessment of plant protection products and other stressors, as well as in restoration programs addressing eutrophication, and in the management of *M. spicatum* as an invasive species. The aim of this study is to describe and provide a data set for model development, refinement, or testing.

## MATERIALS AND METHODS

### Field site and experimental set‐up

This study was conducted at the Sinderhoeve experimental field station, Wageningen University and Research, The Netherlands (51°59ʹ53ʹʹN, 5°45′12ʹʹE), from spring and early summer 2017 until spring and early summer 2019 (including two winters). Environmental conditions were realistic in terms of water and sediment quality and weather (temperature and global radiation under outdoor conditions), and were representative of a temperate climate in the Atlantic–Subatlantic central zone of Europe. One experimental ditch (length 40 m; depth 0.5 m; width 2 m at the sediment surface) was set up for the monitoring of *M. spicatum* populations in plant baskets. The experiments did not involve any addition of toxicants and the ditch was uncontaminated.

The study of the dynamics of the standing crop of *M. spicatum* was performed in 146 plastic pond baskets (width 45 × 45 cm; length 26 cm with an open grid on the sides of the baskets) placed in the experimental ditch. This experimental ditch was cleared of vegetation on 2 May 2017. The bare clay sediment was covered with root cloth on 9 May 2017. On 16 May 2017, the baskets were filled with a 20‐cm layer of natural clay sediment (volume 24.5 L) that originated from a river floodplain. This clay sediment was low in organic matter (3.4%–3.9%), and contained a moisture content of 28.6%–30.3% in fresh sediment samples and a particle distribution of 48.4% below 50 µm and 51.6% above 50 µm (see Supporting Information S1). Pond baskets were then transferred to the ditch, where they were placed on top of the sediment and submerged in water derived from a water basin, which consisted of a mixture of rain water and groundwater with low nutrient content. On 17 May 2017, one bundle of 5–8 *M. spicatum* shoots 15–20 cm in length, provided by Aquaflora, The Netherlands, was planted in each pond basket, with at least two nodes buried in the sediment. Afterwards, the water level was raised to create a water depth of 40–50 cm in the ditch. For sampling purposes, the experimental ditch was virtually divided into three sections. Sampling took place from each of these sections at random. The remaining *M. spicatum* shoots were kept in separate mesocosms and used for short‐term growth experiments.

### Management of experimental ditch

In the experimental ditch, the water level was maintained at a depth of 40–50 cm by inflow from the water basin and outflow to the wastewater basin. During these periods of water inflow and outflow, the ditch was characterized by very slow water movement (residence time of the water >5 days). Despite the root cloth placed on top of the ditch sediment, *Sagittaria sagittifolia* emerged from the original sediment along the banks of the ditch and later also in the center of the ditch. These plants were regularly removed to minimize competition with *M. spicatum*. This management was especially needed in the summer period, when *S. sagittifolia* is known to develop large leaves and flowers.

To promote homogeneous conditions and stimulate water turbulence enhancing carbon uptake (CO_2_ or HCO_3_^−^), six air pumps were installed (Dutch koi Hi‐Blow 8000), which generated gentle bubbling of the upper surface water at these spots.

### Sampling standing crop

The baskets were used to assess population dynamics over two years, but also to determine short‐term seasonal growth rates of *M. spicatum* by placing new shoots into empty baskets at specific time intervals (Table 1). Population dynamics were assessed by harvesting three pond baskets chosen at random from each of the three ditch sections at each sampling date. The frequency of samplings was biweekly in spring, weekly in summer, biweekly in autumn, and monthly in winter. Above‐ground shoots per basket were cut and rinsed with water to remove algae or animals before the shoots were laid out and photographed. The lengths of main and side shoots were measured by hand and added to obtain the TSL. To measure weight, the total above‐ground plant material per basket was placed in preweighed aluminum foil cups. The fresh weight (FW) of biomass was measured after patting the plants with filter paper. Subsequently, samples were dried at 60°C (OECD, 2014b) in an oven for at least 48 h until a constant dry weight (DW) was achieved. Below‐ground biomass, that is, root and rhizome biomass, was measured five times during the study by carefully washing away the sediment from the below‐ground material in a basket. The FW and DW were measured as for above‐ground biomass.

**Table 1:** Population dynamics and four‐week growth rates of *M. spicatum* measured in different set‐ups

Focus	Approach	Endpoints	Period	Factors monitored
Population dynamics	Baskets planted at the start of the experiment and harvested over time	Biomass and shoot length of different baskets over time	May 17–May 19	Continuously: air temp, solar radiation Up to weekly: water temp, pH, conductivity, oxygen, turbidity Up to monthly: nutrients
Growth rates	New top shoots planted in empty baskets at each sampling date and harvested after four weeks	Growth rate of biomass and shoot length in the same basket over time	June 17–Nov 18	

### Measuring growth rates

Short‐term seasonal growth rates of *M. spicatum* shoots without density dependence, but under different temperature and light conditions, were assessed separately in empty baskets planted with new top shoots. For this purpose, the baskets harvested for monitoring population dynamics were replanted with a bundle of three shoots 10 cm in length, which were harvested after four weeks (with some exceptions due to weather conditions). The shoots used for these experiments originated from the same population from which the *Myriophyllum* shoots were taken at the start of the experiment.

On sampling days, photographs were taken of each sample (i.e., per basket) to keep a record of the health and quality of the plant material.

### Measurement of environmental and weather parameters

Electrical conductivity, pH, oxygen, turbidity, and temperature were measured 10 cm below the water surface at the same spots in each of the three sections on each sampling date. Nutrient concentrations were measured every three months, while light in the water column (as photosynthetically active radiation [PAR]) was measured occasionally to assess the overall light conditions in the ditch at the same spot where the other water quality parameters were measured. The PAR was measured using a Li‐Cor Li250A light meter with LWQ 7724 underwater sensor. The organic matter and nutrient (nitrogen and phosphorus) contents of the sediment were measured once to characterize the sediment. Sediment pore water nutrients were measured three times in each of the ditch sections in the summer of 2018.

A weather station (HOBO RX3000 Station*—*CELL‐3G) provided relevant environmental parameters, of which we used solar radiation (at 1.6 m elevation) in W m^−^² and temperature (at 1.5 m) in °C for the analyses. Parameters were measured every 10 min.

### Evaluation

The average specific growth rate r is the change of the natural logarithm of plant FW, DW, or TSL, divided by the time interval.

$$\mu_{i‐j}=(\ln(N_{j})-\ln(N_{i}))/(j-i)$$

where μ_*i*‐*j*_ is the average specific growth rate r from time *i* to *j* and *N_i_*, *N_j_* are the measurement variables at time *i* or *j*.

Growth rates were only calculated from the data obtained in the short‐term growth experiments, since from these experiments data from the same basket at two time points were available. For the population dynamics baskets, the variability between replicates was considered too high to calculate growth rates based on data from different baskets.

### Ratios between the TSL and biomass

Macrophyte models (e.g., Heine et al., 2014, 2015, 2016; Schmitt et al., 2013) usually assume fixed ratios between shoot length and biomass or DW to FW. The monitoring data were used to assess the variability of these ratios across the seasons.

### Correlation and multiple regression analysis

A Pearson correlation analysis was conducted between the mean measured abiotic factors and macrophyte growth rates in terms of TLS, FW, and DW in the different ditch sections. In addition, a multiple regression analysis was conducted to check whether a subset of the environmental factors could predict the observed growth rates. Pore water parameters in the sediment were included in the analyses. Analysis was conducted using SigmaStat for Windows 4‐0 (Systat Software Inc., 2016).

## RESULTS

### Environmental conditions

**Figure 1:**
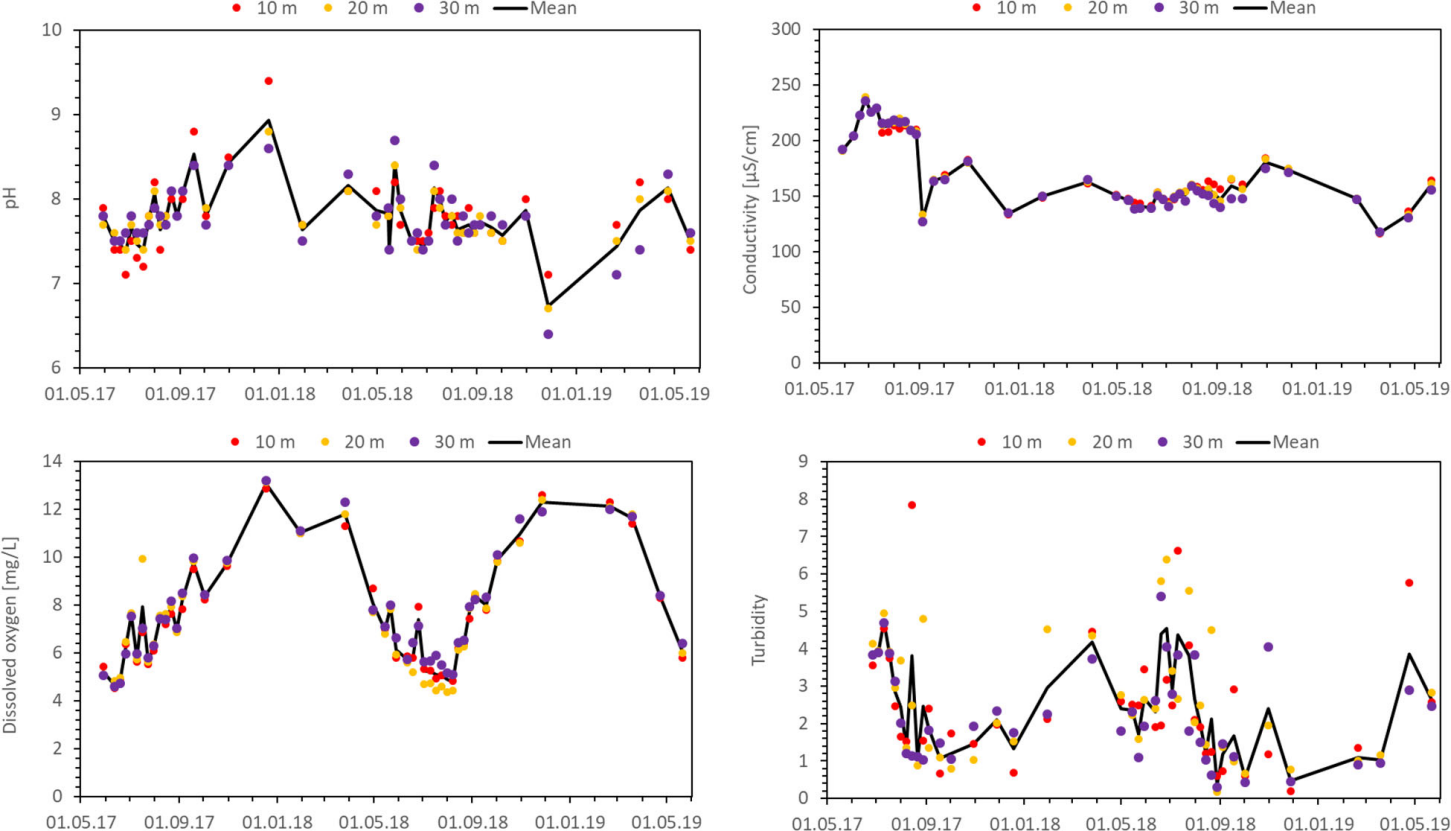
Water parameters of the surface water in the ditch

During the study, weather conditions were representative of a mild sea climate in temperate regions. Air temperature followed the seasonal variation of weather conditions and ranged from a minimum of −8°C in winter to a maximum of 30°C in summer, and comprised one frost period with ice cover (ice cover 18 February to 21 March 2018, Figure 2). Water temperature followed a similar pattern with lower extremes and ranged from 2.7°C in winter up to 25°C in summer (Figure 1). The pH of the ditch water was within the range of 7‒9 (Figure 1 and Supporting Information S1). The observed range of pH values indicates that *M. spicatum* has always had enough CO_2_ or HCO_3_^−^ available for its photosynthesis. Dissolved oxygen (DO) showed a clear seasonal pattern (Figure 1). Conductivity was the highest at the start of the experiment (between 200 and 250 µS cm^−1^) and fluctuated around 150 µS cm^−1^ after the first year. This decrease in conductivity showed that the water was not rich in inorganic carbon. Turbidity was very low (below 8 NTU [nephelometric turbidity units]) on all sampling dates, indicating low amounts of phytoplankton and suspended solids and hence a clear water layer of the experimental ditch. The PAR in the water column varied from a few hundred up to 1200 µmol m^−2^ s^−1^. Nutrient concentrations in the surface water were low, ranging from 0.04 to 0.06 mg L^−1^ for N–NH_4_; 0 to 0.03 mg L^−1^ for N–NO_3_; and 0.02 to 0.025 mg L^−1^ for P–PO_4_. P–PO_4_ showed a higher peak in the second summer, which can be ascribed to decay of plant or algae biomass once the macrophyte‐dominated system developed. Nutrients were probably mainly available to *M. spicatum* via the pore water, where N–NH_4_ ranged from 0.08 to 1.07 mgL^−1^, N–NO_3_ ranged from 0.01 to 0.72 mg L^−1^, and P–PO_4_ ranged from 0.018 to 0.179 mg L^−1^ (see Supporting Information S1).

### Growth dynamics

**Figure 2:**
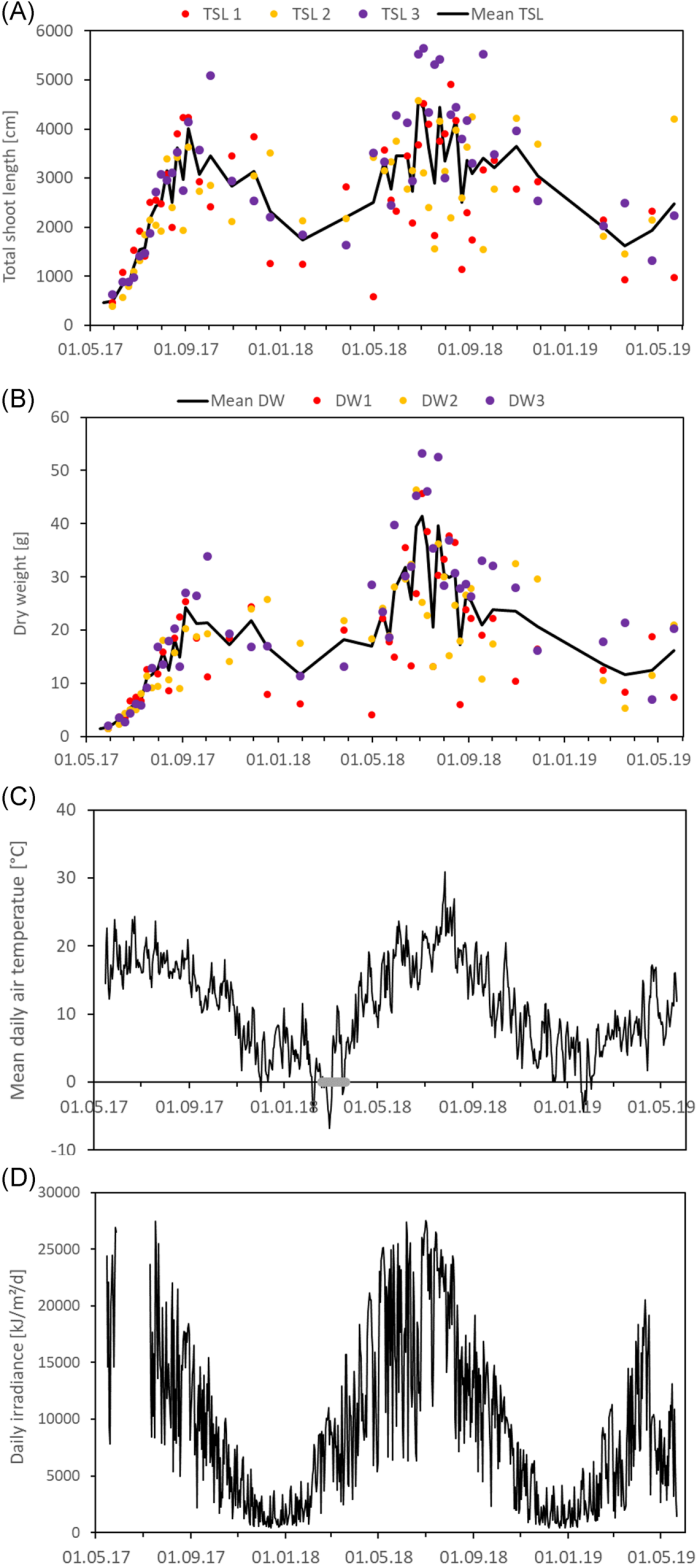
Total shoot length (TSL) (A), dry weight (DW) (B) of *Myriophyllum spicatum*, mean air temperature (C), and daily irradiance (D) over time. The different‐colored dots represent the values from each of the three ditch sections. The mean air temperature is presented in the lower panel (C). The gray line shows the ice cover period, which lasted from February 18 to March 21, 2018

The TSL of *M. spicatum* showed a seasonal pattern, with the highest values in late summer and slight declines in winter (Figure 2). The DW and FW (FW not shown; see Supporting Information) followed seasonal patterns similar to those of the TSL. *M. spicatum* reached a maximum length of 56.4 m per basket and a standing crop above‐ground DW of 53.1 g per basket. However, no major declines in shoot length and above‐ground biomass were observed during the two winter periods. Variability between baskets increased over time, with the maximum variability observed in the summer of 2018, after the experiment had started with very similar low values per basket in 2017. Lower shoot length and biomass values in winter also resulted in reduced variability (Figure 2).

Section 3 of the ditch (the purple dots in Figure 2) on average showed significantly higher TSL, FW, and DW than the other two ditch sections (*p* < 0.05 in one‐sided paired *t*‐test; see Supporting Information S2). This might be caused by the higher NH_4_–N concentration in the water in section 3 resulting in counteracting possible nitrogen limitation. The NO_2_–NO_3_–N and PO_4_–P concentrations showed no significant differences between the sections (see Supporting Information S2).

During the first four months (from 18 May to 4 September 2017), *M. spicatum* growth could be described relatively well by exponential and linear growth functions (Figure 4), with a slightly better fit for the exponential growth model. The average exponential growth rates over these first four months (May–August) were 0.020, 0.023, and 0.023 day^−1^ for TSL, FW, and DW, respectively.

**Figure 3:**
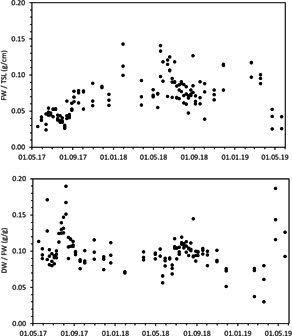
Ratios of fresh weight (FW) to total shoot length (TSL) and dry weight (DW) to fresh weight (FW) of *Myriophyllum spicatum* over time in the population experiment

The DW and FW per TSL were the highest in the second summer season. However, the trends of these ratios over time were not significant (Figure 3). The ratio between DW and the TSL was largely constant, with low coefficients of variation (CV below 30%; Table 2), as was the ratio between FW and DW (CV below 25%; Table 2). The ratio between FW and TSL showed the highest coefficients of variation.

**Figure 4:**
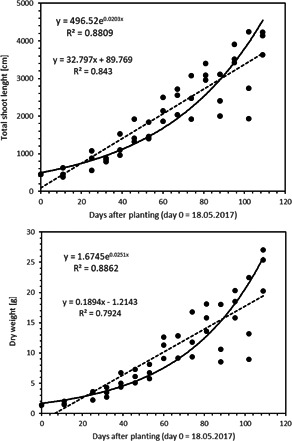
Growth of *Myriophyllum spicatum* over the first four months of the population experiment with fitted linear and exponential functions

**Table 2:** Ratios between fresh weight (FW), dry weight (DW), and total shoot length (TSL) in the population dynamics experiment as presented in Figure 2

	FW/TSL (mg cm^−1^)	DMC = DW/FW (g g^−1^)	DW/TSL (mg cm^−1^)
Mean	70.3	0.0993	6.64
SD	26.7	0.0236	1.96
% CV	38.0	23.8	29.6

Abbreviation: DMC, dry matter content.

As the *M. spicatum* population established from young shoots at the beginning of the experimental period, roots started developing from that point on. Therefore, the ratio between above‐ground and below‐ground biomass was higher than 2 during the first growing season (Table 3). In autumn and in the next year, the average ratios between above‐ground and below‐ground biomass varied between 0.63 and 0.88, indicating an increase in root and rhizome biomass relative to shoots and leaves in established populations of *M. spicatum* (Table 3).

**Table 3:** Ratios between below‐ground and above‐ground biomass

Date	Mean below‐ground dry weight (DW) (g m^−2^)	Mean above‐ground DW (g m^−2^)	Ratio between above‐ground and below‐ground
14 August 2017	6.1	12	2.0
30 October 2017	26	17	0.66
26 June 2018	45	39	0.88
14 August 2018	48	31	0.63
30 October 2018	35	24	0.68

### Periodical growth rates

**Figure 5:**
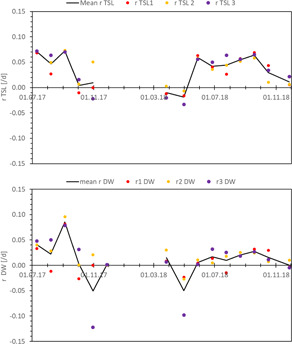
Growth rate (*r*) of total shoot lengths (TSLs) and shoot dry weight (DW) in growth experiments over time

**Figure 6:**
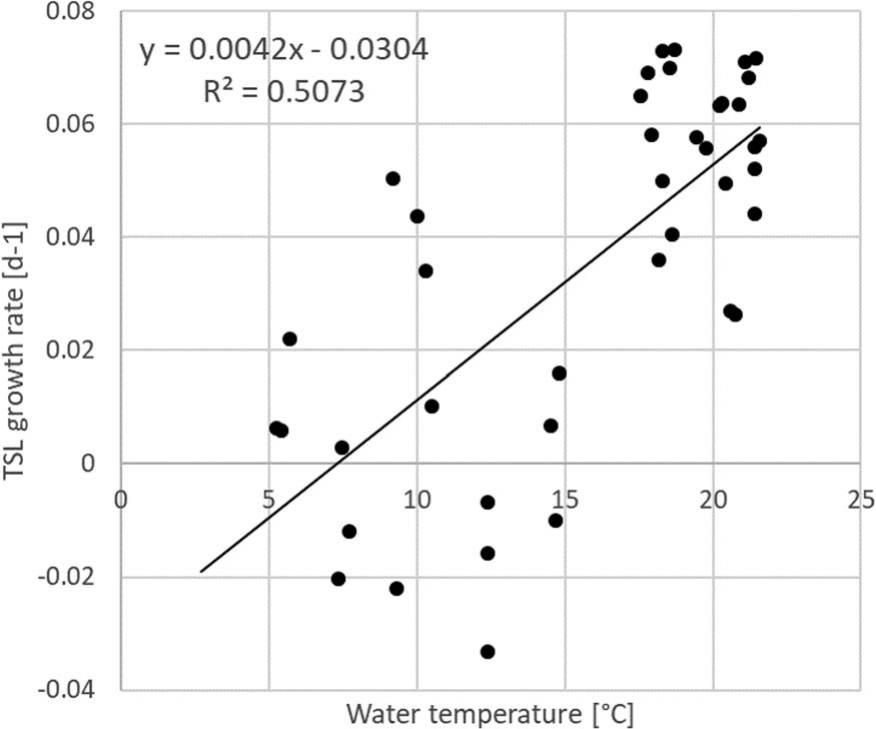
Correlation plots between the growth rate r of total shoot length (TSL) and water temperature. For other correlation plots, see Supporting Information S2 statistics

Periodical growth rates of new shoots over periods usually lasting four weeks reached values up to 0.072, 0.095, and 0.085 day^−1^ for total length, FW, and DW, respectively (Figure 5). These rates showed a seasonal pattern varying from these maximum estimated values per day to zero growth in winter (Figures 5 and 6 and Supporting Information S1).

Pearson correlation analysis revealed some collinearity between the environmental factors (see Supporting Information S2 statistics). Water temperature correlated significantly with air temperature and radiation, ammonium–nitrogen, and turbidity. A very strong negative correlation was found with the concentration of dissolved oxygen (*r* = −0.945) due to the fact that the solubility of oxygen is higher in colder water. The abiotic factor showing the highest linear correlation with TSL growth rate was water temperature (0.713), followed by the mean daily air temperature (0.664), dissolved oxygen (−0.589), ammonium–nitrogen (0.583), radiation (*r* = 0.525), and nitrate–nitrite nitrogen (0.439). No significant correlations were observed between shoot length growth rate and conductivity, pH, turbidity, and phosphate–phosphorus. Correlations between the growth rates of TSL and those of FW and DW were high (0.845 and 0.743, respectively), but among the environmental parameters we measured, only water temperature correlated significantly with the DW growth rate.

In the multivariate regression, water temperature and ammonium and nitrate nitrogen were considered as relevant abiotic factors affecting the growth of *M. spicatum* and showing a significant correlation with TSL growth rate. Air temperature and radiation were not included in the model in view of their high correlation with water temperature. Dissolved oxygen was not used in the model either, since it was considered to be mainly affected by the water temperature and also produced by the macrophytes. Although the pH did not correlate significantly with the growth rate, it was nevertheless included in the regression as an indicator of the CO_2_/HCO_3_^−^/CO_3_ equilibrium in the water, and thus of the availability of different carbon species. The resulting four‐factor model yielded an adjusted *R*² of 0.574 (see Supporting Information S2). However, only pH and water temperature explained a significant proportion of the variability in growth rate (*p* < 0.05). When only these two factors were included in the model, the adjusted *R*² was only slightly lower (0.532) than that for the four‐factor model.

Since we only had nine values from pore water, we did not evaluate this. The values were higher than those in surface water, especially for NH_4_–N (see Supporting Information S1).

## DISCUSSION

### Environmental conditions

The experimental ditch system used in this study is representative of shallow, macrophyte‐dominated freshwater ecosystems in the temperate region in Europe (Arts et al., 2006; Cuppen et al., 1997). The conditions in this ditch enabled very good growth of submerged macrophytes, since the maximum biomass values and growth rates are coherent with published studies (e.g., Grace & Wetzel, 1978). The water layer was clear, as indicated by the low turbidity (see Supporting Information S1; we measured nephelometric turbidity, i.e., an index of light scattering by suspended particles like algae or sediment), so there was little competition with phytoplankton. Nutrients were mainly available in the sediment and available for macrophytes by root uptake, thereby contributing to a clear water layer. Water depth was low and light penetrated down to the bottom (so light was not limiting); the range of PAR was determined by radiation and overcast, but this is not uncommon for shallow mesocosms (Hanson et al., 2003). The pH never reached a value of 10, so we never had a situation where carbon was not available for aquatic macrophytes. The finding that pH was a significant parameter in the regression model can be explained by this factor being a surrogate for the CO_2_–HCO_3_^−^–CO_3_ equilibrium.

### Variability in time and space

The TSL, FW, and DW varied from one sampling to the next, so no meaningful growth rates could be calculated from the values in different baskets. Although the experiment started with identically prepared plant baskets, filled with well‐mixed sediment and planted with similar shoots originating from one population of *M. spicatum*, the variability in growth may have been caused by the location in the ditch. The highest values of TSL and biomass were, on average, found in section 3 of the ditch, where the inlet of fresh water was situated for the purpose of sustaining the water level. This higher growth rate in section 3 can be explained by a higher availability of nitrogen as the ammonium–nitrogen concentration in the water was significantly higher in section 3 than in the two other sections. The molar ratios of N to P in both water and pore water are frequently below 20:1 and 25:1, suggesting the nitrogen limitation of the macrophytes under these circumstances. This limitation has been compensated for by the renewal of water in the ditch.

Internal competition with surrounding plants in other baskets or outside the baskets at the ditch side could have played a role, depending on how many baskets in the neighboring area had already been harvested.

### Correlation between abiotic factors and growth rates

In our data set, water temperature and pH explained more than half of the variability in growth rates (53.2%). The pH alone did not correlate significantly with the growth rate, but was found to be more relevant in combination with water temperature than nitrogen or phosphorus concentrations in the water layer. However, pH might affect the growth of the plants as an indicator of the available CO_2_ in the water, but it is also affected by the photosynthesis of algae and macrophytes (Maberly & Spence, 1983). It should be noted that the mean of the environmental variables measured at the start and end of the growth intervals (usually four weeks) is only a rough estimation of the variable environmental conditions during the interval. However, the available data indicate that under the conditions prevailing in the ditch, the concentrations of nitrogen and phosphorus in the water were less relevant than the available carbon species. The limited data set for the pore water suggests that phosphorus could be a significant predictor of the growth of *M. spicatum*. An explanation for the lack of correlation between PO_4_‒P and growth of shoots could be limitation by nitrogen.

### Standing crop maximum biomass

Our maximum values for standing crop were 56.4 m per basket for total above‐ground length and 53.1 g per basket for standing crop above‐ground DW. We wanted to compare the maximum standing crop biomass found in our study with maximum biomass data from lake, pond, or ditch studies, but we found that quantitative data were only available from North America, where *Myriphyllum spicatum* is an invasive species. Smith and Adams (1986) found a maximum standing crop biomass of 130 g m^−2^ DW in lake Wingra, Wisconsin. Madsen (1997) found a biomass up to 1500 g m^−^² in experimental ponds of 1 m depth. The highest standing crop biomass, 2283 g DW m^−2^, has been found in Fish Lake, Wisconsin (Best & Boyd, 1999; Budd et al., 1995; Carpenter, 1980). For comparison, our maximum standing crop was 262 g m^−2^ DW when recalculating the maximum value for a basket to a m^2^ value supposing an equal density of plant biomass per m^2^ as per basket of 0.2025 m^2^. This maximum standing crop is probably an overestimation as a result of the free space around a basket. Although seasonal biomass maxima may vary considerably over time and space (Best & Boyd, 1999), we do not believe that the extremely high value of more than 2 kg DW m^−2^ is relevant for risk assessment, as it is important to consider the biomass production and standing crop in the biogeographical region where *M. spicatum* is native. Multiseasonal time series from temperate regions are lacking, as the data from Madsen (1997) were collected in Texas (USA), representing a climate with hot summers and cold winters. This means that our data set from temperate regions is ecologically relevant and fills an important gap.

Studies in temperate climates have described mostly one (as in our study), but occasionally also two biomass peaks per year (Best & Boyd, 1999). Best and Boyd (1999) is in fact the only study among the references below to report two biomass peaks. These double peaks are likely due to multiple flowering periods (Titus et al., 1975). Climate (latitude), water transparency, and depth distribution of the macrophytes exert a strong influence on the maximum biomass achieved (Best & Boyd, 1999; Duarte & Kalff, 1987). In our study, the flowering and fruiting period of *M. spicatum* lasted from 12 June to 30 October 2017 and from 30 April 2018 to 30 October 2018. Flowering started again in April 2019. Best and Boyd (1999) reported that flowering usually coincides with peak biomass, but this was not the case in our experiment, where the flowering and fruiting period covered several months. The trigger for the production of biomass is the temperature. We found that growth rates were higher above 15°C, while at temperatures below 15°C, growth rates varied and could be explained by other factors. These findings are in agreement with Smith and Barko (1990) and Nichols and Shaw (1986), who stated that a temperature exceeding 15°C stimulates the growth of *M. spicatum*. In general, the growth period lasts from spring until autumn, and our study showed that this period can last until late autumn.

The biomass of *M. spicatum* is unevenly distributed over the water column, with typically more than 60% concentrated in the upper water layers (Best & Boyd, 1999). During growth, especially in deeper waters, the percentage of total plant length and biomass comprised of foliated stems decreases over time (Budd et al., 1995). Water depth influences the morphology of the plants, as those growing in deep water are long and thin, while those growing in shallow water are shorter and more robust, with larger relative amounts of foliage (Budd et al., 1995; Strand & Weisner, 2001). Early in spring, *M. spicatum* plants grow from established rootstocks and develop mainly short shoots going more or less straight up to the surface, then growing along the surface to form a canopy in areas of dense growth (Adams et al., 1974). Meanwhile, leaves are sloughed off from the lower stems. Thus, by mid‐ and late summer, most of the active photosynthetic tissues (leaves) are located within the canopy near the water surface, whereas leafless stems predominate below the canopy (Adams et al., 1974). The maximum rhizome biomass in the baskets in our study was 14.5 g per basket, which—when considered per m^2^—is very close to the values reported in the literature (a maximum root value of 50 g m^−2^ and an average value of 29 g m^−2^ measured in Lake Wingra in 1977; Smith & Adams, 1986).

We did not observe large‐scale die‐back of the plants during winter, probably due to two relatively warm winters during the experimental period. This means that a significant above‐ground standing crop was present in the experimental ditch in winter as well. The literature reports that *M. spicatum* most frequently overwinters as root crowns and/or lower shoots attached to the rhizome system (Grace & Wetzel, 1978; Madsen, 1997) and may maintain considerable winter biomass (Madsen, 1997; Smith & Barko, 1990; Stanley et al., 1976). This is consistent with the results from our monitoring study. Variations in this annual pattern result from differences in climate, water clarity, and rooting depth (Smith & Barko, 1990). Another explanation might be the absence of any grazing pressure by water fowl. The presence of a significant above‐ground standing crop of *M. spicatum* might be representative of warmer climate conditions expected to occur more often in temperate regions in near future.

### Growth rates of *M. spicatum* under outdoor conditions

The maximum biomass‐based growth rates of *M. spicatum* shoots found in our study in the first summer (0.085 day^−1^ for DW biomass; see Supporting Information S1) were of the same order of magnitude or higher than those found in 21‐day single species laboratory test that included a sediment in the test design (0.056 ± 0.007 day^−1^ for biomass; Knauer et al., 2006), as well as in biomass‐based tests reported in a 20‐day outdoor mesocosm study (0.068 ± 0.014 day^−1^; Knauer et al., 2008), and in a stream mesocosm study (Wieczorek et al., 2017; maximum mean growth rate for total shoot DW 0.066 ± 0.010 day^−1^). If we consider the endpoint TSL, we observed a maximum growth rate of 0.072 day^−1^. This is higher than those reported from laboratory single species tests (0.050 ± 0.014 day^−1^; Knauer et al., 2006) and from a mesocosm study (0.039 ± 0.006 day^−1^; Knauer et al., 2008), and lies in the same range as that reported by Wieczorek et al. (2017), who found a maximum growth rate of 0.078 ± 0.007 day^−1^. The lower values reported by Knauer et al. (2006) refer to a water‐only *M. spicatum* study lacking any sediment. Nielsen and Sand‐Jensen (1991) reported a growth rate for *M. spicatum* of 0.046 ± 0.004 day^−1^ under optimum carbon availability conditions in the laboratory. Knauer et al. (2006) presented a table with growth rates for the TSL of *M. spicatum* varying from 0.050 to 0.143 day^−1^ in laboratory studies and microcosms. The upper value was confirmed by other laboratory studies (range = 0.1–0.15 day^−1^) and is about twice the value found in the current study. Thus, the growth conditions in our study were in the range of those in laboratory and mesocosm studies, despite the longer time frame of our study.

The main difference between our study and *M. spicatum* laboratory studies is that those are performed under optimum and constant conditions (OECD, 2014b), with a fixed daily rhythm of dark and light periods and addition of nutrients to the artificial sediment. The aim of our experimental ditch study was to investigate the growth of *M. spicatum* under realistic outdoor conditions, including seasonal variations of environmental parameters over a longer time frame. Over the first four months of our experiment, the average growth rate was 0.020 day^−1^ for TSL and 0.023 day^−1^ for FW and DW, which is about half of the minimum growth rate required for a valid OECD 239 test (doubling TSL and FW over 14 days, corresponding to *r* = 0.050 day^−1^) and lower than the control growth rates that can be reached in such tests (up to 0.15 day^−1^, Andreas Solga, Bayer AG, personal communication). However, the maximum growth rates over 28 days in the growth rate experiments in our ditch were about 0.1 day^−1^, which is close to the values usually found in the standardized laboratory OECD test. It is apparent that macrophytes in the field, after having shown an exponential or linear growth phase, will reach a plateau; thus, growth rates will not further increase, but decrease with time.

### Regulatory context

The aim of this study was to generate ecologically relevant, long‐term data series for testing, refinement, and/or as input parameters for *M. spicatum* population models. Such models—in combination with TK–TD modeling—might play an important role in the risk assessment when extrapolation of the effects of, for example, herbicides from laboratory tests to populations under field conditions and longer time frames is required. In temperate regions, it is important to take seasonal variation in macrophyte growth into account in this context, and long‐term data series covering several seasons are necessary to calibrate or validate models as being fit for purpose for such extrapolations. Many herbicides enter aquatic systems during spring, when temperatures and light conditions are just starting to become suitable for macrophyte growth and *M. spicatum* plants are depleted in their carbohydrates (Madsen, 1991). Similarly, herbicides are also often used in autumn, when the biomass of *M. spicatum* is decreasing and carbohydrates are building up in the plant (Madsen, 1991). Models might facilitate the extrapolations over several seasons. This study presents a data set on the dynamics of *M. spicatum* in a ditch checked over two years under temperate conditions, including two relatively warm winters. The conditions are realistic for a wide geographical region with temperate climate. To complement this, similar data sets obtained under different conditions would be useful. The environmental conditions prevailing during our study are provided in the Supporting Information, and can be used as model input, while the resulting dynamics predicted by the model can be compared with the field observations. In addition, the data from our short‐term experiments that started at different times over the year can be used to test the modules of models describing the dependence of growth on environmental factors, especially temperature and radiation.

## CONCLUSIONS

− This study has generated a time series of seasonal dynamics for the growth of *M. spicatum* over two years under environmental conditions found in temperate regions.
− *M. spicatum* showed a clear seasonal pattern of biomass and shoot length and of their variability (increasing in summer and decreasing in winter).
− Over the first four experimental months in summer, exponential functions yielded a better fit for the growth of *M. spicatum* than linear growth functions.
− During the mild winters for temperate regions that prevailed in our study period, above‐ground biomass declined only slightly.
− Multiple regression modeling revealed that water temperature and pH (the latter as a surrogate for the available carbon species) explained a significant proportion of the variability in *Myriophyllum* growth rates (*p* < 0.05), while the phosphate–phosphorus concentration explained the largest proportion of the variability for sediment pore water.
− The data set can be used to develop and test population models for *M. spicatum*.

## CONFLICT OF INTEREST

The Dutch TKI programme requires the open‐access publication of all results and all data. This was the intention of the project right from the beginning. The authors listed immediately follow certify that they have no conflicts of interest to declare: Gertie H. P. Arts, J. van Smeden, M. F. Wolters, J. D. M. Belgers, A. M. Matser, U. Hommen, The authors listed immediately follow are members of CropLife Europe expert groups and are employed by commercial companies: E. Bruns, S. Heine, A. Solga, S. Taylor. The peer review for this study was managed by the Editorial Board without the involvement of U. Hommen.

### OPEN DATA BADGE

This article has earned an Open Data Badge for making publicly available the digitally shareable data necessary to reproduce the reported results. The data are available at https://doi.org/10.4121/15368442. Learn more about the Open Practices badges from the Center for Open Science: https://osf.io/tvyxz/wiki.

## Supporting information

This article includes online‐only Supporting Information.

The Supporting Information includes three files: S1. Data and figures; S2. Statistics; S3. Weather data.

## Data Availability

The experimental data sets covering 2 consecutive years, the regression sheets, correlation matrices, statistical reports, and weather data are available through an online repository: 4TU. Research data. https://doi.org/10.4121/15368442.
